# Risk factors for mortality after hospitalization for suicide attempt: results of 11-year follow-up study in Piedmont Region, Italy

**DOI:** 10.1007/s00127-023-02544-7

**Published:** 2023-08-08

**Authors:** Emina Mehanović, Gianluca Rosso, Gian Luca Cuomo, Roberto Diecidue, Giuseppe Maina, Giuseppe Costa, Federica Vigna-Taglianti

**Affiliations:** 1 Department of Neurosciences ‘Rita Levi Montalcini’, University of Turin, Turin, Italy; 2 Piedmont Centre for Drug Addiction Epidemiology, ASL TO3, Grugliasco, Turin, Italy; 3 Psychiatric Unit, San Luigi Gonzaga University Hospital, Orbassano, Turin, Italy; 4 Department of Clinical and Biological Sciences, University of Turin, Orbassano, Turin, Italy; 5 Department of Translational Medicine, University of Eastern Piedmont, Novara, Italy

**Keywords:** Suicide attempt, Mortality, Risk factors, Cox regression, Italy

## Abstract

**Purpose:**

Suicide attempters are at high risk of premature death, both for suicide and for non-suicidal causes. The aim of this study is to investigate risk factors and temporal span for mortality in a cohort of cases admitted to hospital for suicide attempt.

**Methods:**

The cohort included 1489 patients resident in Piedmont Region, North West of Italy, who had been admitted to hospital or emergency department for suicide attempt between 2010 and 2020. Cox regression models were used to identify risk factors for death. The final multivariate model included gender, age, area deprivation index, family composition, psychiatric disorders, malignant neoplasms, neurological disorders, diabetes mellitus, cardiovascular diseases, chronic obstructive pulmonary disease, and intracranial injury or skull fracture.

**Results:**

During the observation period, 7.3% of patients died. The highest mortality was observed within the first 12 months after suicide attempt, and remained elevated for many years afterwards. Male gender, older age, high deprivation index of the census area, single-parent family, mood disorders, malignant neoplasms, diabetes mellitus and intracranial injuries or skull fracture were independent predictors of death. Risk factors for natural and unnatural causes of death were also identified.

**Conclusions:**

The mortality risk of suicide attempters is very high, both in the months immediately following the attempt and afterwards. The identification of high-risk groups can help to plan outpatient care following the hospital discharge. Our findings urge the need to design strategies for the assistance and care of these patients at long term in order to reduce the unfavourable outcomes.

## Introduction

Suicide attempters are at high risk of premature death, both for suicide and for non-suicidal causes [1–13]. Mortality rates vary from 1.2 to 37.6% in studies covering a follow-up period of 1–32 years [1–3, 5, 7, 9, 10, 12–19]. The risk of death is highest within the first year after the episode of suicide attempt and remains elevated for many years afterwards [1, 2, 6, 7, 10, 11, 13, 16, 17, 20, 21].

The burden of suicide attempt is difficult to estimate due to underreporting and lack of standard reporting procedures in many countries. The prevalence of lifetime suicide attempt was estimated as 1.8% in 2001–2003 in Europe [22], and 0.3–4.2% in 2001–2007 worldwide [23–25]. In more recent years, an increasing trends of suicide attempt rates have been reported in some countries [26–30].

The identification of sociodemographic and clinical risk factors predicting premature mortality is of paramount importance to address high-risk subgroups and target them with appropriate health care and prevention interventions. A number of risk factors for mortality have been documented in prospective studies, i.e. male gender, older age, living alone, being single, lower education, income area/socioeconomic status, unemployment, retirement, violent method of suicide attempt and repetition of attempt [1–7, 9, 11, 13–18, 20, 31–34]. Psychiatric disorders and physical illnesses co-occur commonly and represent a noteworthy risk factor for increased mortality among suicide attempters, particularly in the presence of both conditions [2, 4, 5, 21, 22, 28, 31, 35–37]. Among psychiatric disorders, anxiety, depressive and bipolar disorder, schizophrenia, alcohol and drug abuse, and personality disorder are the most recognized co-existing diagnoses associated with mortality risk, and when co-occurring can significantly reduce the temporal interval from first episode of suicide attempt to death [3, 4, 6, 9, 13, 20, 21, 33, 37].

Most studies on risk factors for mortality following suicide attempt were conducted in North-Europe where the rates of suicidal behaviours are particularly high. Due to different prevalence rates and context, studies investigating the phenomenon in South-Europe are needed.

The aim of this study is to investigate risk factors and temporal span for mortality in a cohort of cases admitted to hospital or emergency department for suicide attempt between 2010 and 2020 in Piedmont, a North–West Italian Region with high rates of suicidal behaviour [38].

## Methods

### Study sample

The cohort included 1489 patients resident in Piedmont, a region of 4.4 million inhabitants in North West of Italy, who had been admitted to hospital or emergency department (ED) for suicide attempt between 1st January 2010 and 31st December 2020.

Suicide attempts were defined according to the International Classification of Diseases, 9th Revision (ICD-9), as recorded at hospital or emergency department discharge: suicide and self-inflicted injury (E950-E959), injury undetermined whether accidentally or intentionally inflicted (E980-E989), and suicidal ideation (V62.84).

All patients who had at least one episode of suicide attempt during the study period were included in the cohort. If there was more than one episode of suicide attempt, the first episode occurred during the study period was used to define the patients’ date of entry into the cohort. In order to allow record-linkage of administrative and health data, only subjects who resided in Piedmont at the time of ED or hospital discharge were included in the cohort. Patients aged 12–74 years were included in the study. Patients were followed until death, emigration, or the end of the study. Follow-up started from the date of discharge from hospital or ED. All patients were followed up longitudinally for up to 11 years, i.e. follow-up ended on 31st December 2020.

### Data collection

Information on the patients in the study was collected through a record-linkage of the administrative and health data available in the Piedmont Longitudinal Study. Linkage of the data archives was done using an anonymous identification code under the frame of the National Statistical Act that legitimates the use and linkages of data for scientific purposes without the need of an Ethical Committee approval.

The sources of data were: 2011 Population Census of the Piedmont Region and the NHS Regional Population Registry for sociodemographic characteristics; and the hospital discharges and emergency department databases for health-related conditions and diagnoses. Information on all-cause mortality (dead/alive) was extracted from the NHS Regional Population Registry available for the period 2010–2020. Information on cause-specific mortality was obtained from the Mortality Register of the Piedmont Region available for the period 2010–2018.

### Measures

The following sociodemographic characteristics were extracted: gender, date of birth, age at the index episode of suicide attempt, deprivation index of the census area, marital status, education, occupation and family composition. Area deprivation index was based on five conditions describing social and material deprivation, measured at the census section level: % of population with low education, % of unemployed, % of population living in rented houses, in crowded households, in single-parent families. The area deprivation index was then categorized in quintiles (1st = lowest to 5th = highest deprivation) [39].

Psychiatric disorders were coded according to the International Classification of Diseases, 9th Revision (ICD-9): schizophrenia [schizophrenic disorders (295–295.95), other non-organic psychosis (297–298.9)]; bipolar disorders (296.0–296.16, 296.4–296.81, 296.89); personality disorders (301–301.9); depressive disorders (296.2–296.36, 300.4, 311); drug and alcohol dependence (291–292.9, 303–305.93); anxiety disorders (300.0–300.3, 300.5–300.9); adjustment disorders [adjustment reaction (309–309.9), acute reaction to stress (308–308.9)]; dementia [dementia (290–290.9), other mental disorders due to organic condition (293–294.9)]. A mutually exclusive categorisation of psychiatric diagnoses was used, applying the following hierarchy: schizophrenia, bipolar disorder, personality disorder, depressive disorder, drug and alcohol dependence, anxiety disorder, adjustment disorder, others (dementia and other mental disorder due to organic condition), none.

The following physical illnesses were studied (ICD-9 codes): malignant neoplasm (140–208.91); neurological disorders [Alzheimer’s disease (331.0), Parkinson’s disease (332–332.1), extrapyramidal diseases and myelopathies (333–336.9), disorders of autonomic nervous system (337–337.9), multiple sclerosis (340), epilepsy and recurrent seizures (345–345.91), migraine (346–346.91), disorders of peripheral nervous system (350–359.9)]; diabetes mellitus (250–250.93); cardiovascular diseases [hypertension (401–405.99), ischemic heart disease (410–414.9), cerebrovascular disease (430–438.9)]; chronic obstructive pulmonary disease (490–496); dorsopathies (720–724.9); intracranial injury or skull fracture (850–854.19, 800–804.99). Physical illnesses were analysed as dichotomous variables (yes/no).

Causes of death were classified according to the International Statistical Classification of Diseases and Related Health Problems, 10th Revision (ICD-10), and for the scope of this study were grouped in natural deaths [neoplasms (C00-D48); endocrine, nutritional, and metabolic diseases (E00-E90); mental and behavioural disorders (F00-F99); diseases of the nervous system and sense organs (G00-H95); diseases of the circulatory system (I00-I99); diseases of the respiratory system (J00-J99)]; and unnatural deaths [accidents (V01-X59); intentional self-harm (X60-X84); assaults (X85-Y09)].

### Statistical analysis

The primary outcome under study was all-cause death (yes/no).

Secondary analyses were conducted on cause-specific mortality (natural and unnatural).

Descriptive statistics were used to describe the study population by gender. Kaplan–Meier survival analysis was used to plot temporal patterns of death after ED or hospital discharge for suicide attempt. The curves of the subgroups were compared through log-rank test. For each patient in the cohort, person-years of survival were calculated starting from the date of discharge from ED or hospital of the index episode to the date of death or the end of follow-up.

Gender, age, area deprivation index, marital status, education, family composition, psychiatric disorders and physical illnesses were evaluated as risk factors for death in Cox regression models.

Unadjusted Cox regression models were performed to identify risk factor independently from other covariates. Collinearity among variables was checked. The non-collinear and statistically significant variables were then selected to build the multivariate model. Due to correlation between marital status and family composition (*r* = 0.7), only family composition was included in the final model. However, sensitivity analysis was performed with marital status included in the model instead of family composition. Some variables had several missing data, therefore, to minimize the reduction of cases in the regression models, the additional level “Missing” was created. Analyses of Schoenfeld residuals and Kaplan–Meier were performed to test proportional hazard assumptions, before building the final multivariate Cox regression model. The proportional hazard assumption was satisfied for all variables except for psychiatric disorders and chronic obstructive pulmonary diseases in the model focused on unnatural deaths. Since the Kaplan–Meier survival curves showed violation only in the first months of the follow-up, both factors were added in the Cox regression model. However, results of this model should, therefore, be considered in light of this limitation.

The final Cox model included: gender, age, area deprivation index, family composition, psychiatric disorders, malignant neoplasms, neurological disorders, diabetes mellitus, cardiovascular disorders, chronic obstructive pulmonary disease and intracranial injury or skull fracture. As regards psychiatric disorders, the diagnoses included in the category of “other disorders” (personality disorder, drug and alcohol dependence, anxiety disorder, adjustment disorder, dementia and other disorders due to organic conditions) showed protective effect compared to no disorder at all in the preliminary analysis. Therefore, in the multivariate analysis, these two groups were merged as reference level, and psychiatric disorders were re-grouped into three categories: none/other disorders; schizophrenia; and mood disorders (bipolar and depressive disorders). The multivariate Cox regression models assessing risk factors for natural and unnatural causes of death were run on the restricted sample of patients entering the cohort between 2010 and 2018.

All the analyses were performed by using STATA 16 statistical software [40].

## Results

### Descriptive statistics

Sociodemographic and clinical characteristics of the study sample are shown in Table 1.

**Table 1:** Characteristics of patients admitted to hospital or ED for suicide attempt between 2010 and 2020, in Piedmont Region, Italy, by gender

Characteristics	Suicide attempters						
	Overall (*N* = 1489)		Males (*N* = 672)		Females (*N* = 817)		*P*
	*n*	%	*n*	%	*n*	%	
Age (years)							
Mean ± SD	38.6 (17.4)		42.1 (16.2)		35.7 (17.8)		** < 0.001**
Age groups (years)							
12–14	122	8.2	22	3.3	100	12.2	** < 0.001**
15–29	388	26.1	149	22.2	239	29.3	
30–44	353	23.7	182	27.1	171	20.9	
45–59	449	30.1	222	33.0	227	27.8	
60–74	177	11.9	97	14.4	80	9.8	
Area deprivation index							
1 quintile (lowest deprivation)	282	18.9	139	20.7	143	17.5	0.167
2 quintile	254	17.1	112	16.7	142	17.4	
3 quintile	255	17.1	105	15.6	150	18.4	
4 quintile	271	18.2	115	17.1	156	19.1	
5 quintile (highest deprivation)	226	15.2	98	14.6	128	15.7	
Missing	201	13.5	103	15.3	98	12.0	
Marital status							
Married	390	26.2	192	28.6	198	24.2	**0.037**
Unmarried	706	47.4	293	43.6	413	50.6	
Separated/Divorced	165	11.2	74	11.0	91	11.1	
Widowed	36	2.4	14	2.1	22	2.7	
Missing	192	12.9	99	14.7	93	11.4	
Education							
University	64	4.3	29	4.3	35	4.3	** < 0.001**
High school	236	15.9	107	15.9	129	15.8	
Middle school	599	40.2	299	44.5	300	36.7	
Elementary school	175	11.7	90	13.4	85	10.4	
None	194	13.0	43	6.4	151	18.5	
Missing	221	14.8	104	15.5	117	14.3	
Occupation							
Employed	486	32.6	285	42.4	201	24.6	** < 0.001**
Unemployed	117	7.9	56	8.3	61	7.5	
Retired	126	8.5	69	10.3	57	7.0	
Other^a^	278	18.7	91	13.5	187	22.9	
Missing	482	32.4	171	25.5	311	38.1	
Family composition							
Couples with children	656	44.1	276	41.1	380	46.5	** < 0.001**
Couples without children	157	10.5	74	11.0	83	10.2	
Single parent	227	15.3	84	12.5	143	17.5	
Single	221	14.8	125	18.6	96	11.8	
Other^b^	26	1.8	9	1.3	17	2.1	
Missing	202	13.6	104	15.5	98	12.0	
Psychiatric disorders							
Schizophrenia	423	28.4	193	28.7	230	28.2	**0.001**
Bipolar disorder	98	6.6	40	6.0	58	7.1	
Personality disorder	319	21.4	134	19.9	185	22.6	
Depressive disorder	250	16.8	103	15.3	147	18.0	
Drug and alcohol dependence	80	5.4	53	7.9	27	3.3	
Anxiety disorder	67	4.5	24	3.6	43	5.3	
Adjustment disorder	63	4.2	24	3.6	39	4.8	
Other^c^	9	0.6	5	0.7	4	0.5	
None	180	12.1	96	14.3	84	10.3	
Malignant neoplasms	88	5.9	46	6.8	42	5.1	0.165
Neurological disorders	204	13.7	78	11.6	126	15.4	**0.033**
Diabetes Mellitus	82	5.5	48	7.1	34	4.2	**0.012**
Cardiovascular diseases	270	18.1	162	24.1	108	13.2	** < 0.001**
Chronic obstructive pulmonary disease	89	6.0	45	6.7	44	5.4	0.288
Dorsopathies	318	21.4	147	21.9	171	20.9	0.658
Intracranial injury/Skull fracture	196	13.2	103	15.3	93	11.4	**0.025**

*SD* standard deviation, *P* *p*-value

^a^Other: student, housewife, other conditions

^b^Other: families with two or more nucleus or other relatives

^c^Other: dementia and other mental disorders due to organic condition

From 2010 to 2020 in Piedmont Region, a total of 1489 patients aged 12–74 years were admitted to hospital or ED with a diagnosis of first suicide attempt, 817 (54.9%) females and 672 (45.1%) males. The mean age of the patients was 38.6 (± 17.4), males being older than females (42.1 vs. 35.7, *p* < 0.001). About 34% were adolescents and young adults aged 12–29 years. No differences were observed for area deprivation index. Sixty-one percent of patients were alone (unmarried, separated or divorced, widowed), 64.4% among females and 56.7% among males (*p* < 0.037). About 40% of patients had middle school education level, a greater proportion of males had middle (44.5% vs. 36.7%) and elementary school education (13.4% vs. 10.4%), whilst no education was more frequent among females (18.5% vs. 6.4%) (*p* < 0.001). About one third of patients were employed (42.4% of males vs. 24.6% of females) and 8.5% were retired (10.3% of males vs. 7.0% of females) (*p* < 0.001). A higher proportion of females lived in families defined as couples with children and single-parent families (46.5% vs. 41.1%, and 17.5% vs. 12.5%, respectively), whereas the proportion of singles was higher among males (18.6% vs. 11.8%) (*p* < 0.001).

Eighty-eight percent of patients had a record of psychiatric disorders, with a greater proportion of males being diagnosed with drug and alcohol dependence (7.9% vs. 3.3%), and females with personality disorder (22.6% vs. 19.9%), depressive disorder (18.0% vs. 15.3%) and anxiety disorder (5.3% vs. 3.6%) (*p* = 0.001).

A greater proportion of males was diagnosed with diabetes mellitus (7.1% vs. 4.2%, *p* = 0.012), cardiovascular diseases (24.1% vs. 13.2%, *p* < 0.001) and intracranial injury or skull fracture (15.3% vs. 11.4%, *p* = 0.025), whereas females with neurological disorders (15.4% vs. 11.6%, *p* = 0.033).

During the observation period, 7.3% of patients died (10.0% of males and 5.0% of females, *p* = 0.001).

### Survival analysis

**Fig. 1:**
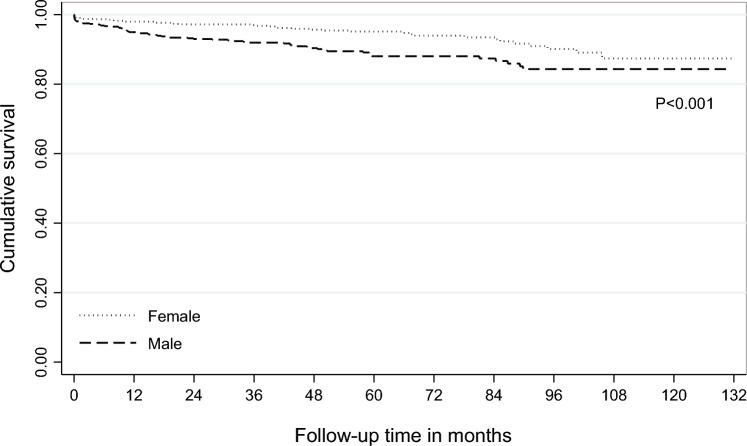
Kaplan–Meier survival curve for patients admitted to hospital or ED for suicide attempt between 2010 and 2020, in Piedmont Region, Italy, by gender

**Fig. 2:**
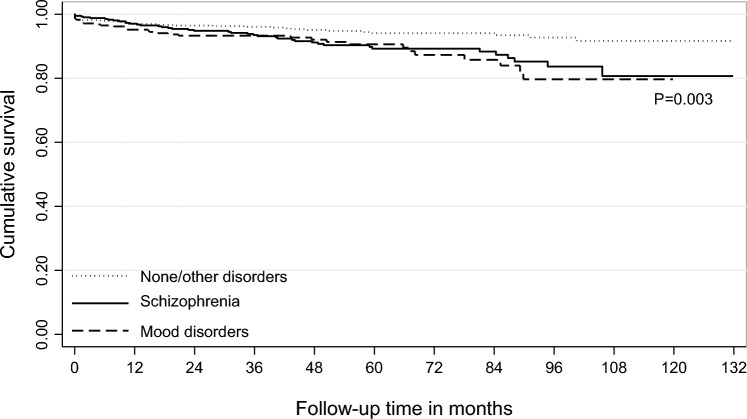
Kaplan–Meier survival curve for patients admitted to hospital or ED for suicide attempt between 2010 and 2020, in Piedmont Region, Italy, by psychiatric diagnosis

The Kaplan–Meier survival curves by gender and psychiatric disorders are shown in Figs. 1 and 2.

Mortality was highest during the first 12 months after the index suicide attempt: 45.4% of deaths occurred in the first year, 14.8% in the second year, and the others were sparse over the following 7 years. No deaths occurred in the last 2 years of the observation period.

Through the entire follow-up, the survival rate was significantly lower for males than females (log rank *p* < 0.001); 33 males and 16 females died in the first 12 months (Fig. 1).

The survival rate was significantly lower for patients diagnosed with schizophrenia and mood disorders than those with no disorders or other disorders (log rank *p* = 0.003) (Fig. 2). This was observed also when looking at natural causes of death (data not shown).

### Cox regression models for all-cause mortality

In unadjusted Cox regression models, the risk of death was significantly higher for males, progressively increased with age, and was significantly higher for widowed, couples without children, single-parent family, and single. The risk of death was significantly associated with schizophrenia, mood disorders, malignant neoplasms, neurological disorders, diabetes mellitus, cardiovascular diseases and chronic obstructive pulmonary diseases. The association of high area deprivation index and intracranial injury or skull fracture with the risk of death was marginally significant (Table 2).

**Table 2:** Risk factors for all-cause death (2010–2020): unadjusted Cox regression models

Characteristics	Death	PY	Crude HR (95% CI)	*P*
Gender				
Female	41	40,340.45	1	
Male	67	33,307.89	1.99 (1.35–2.93)	**0.001**
Age (years)	108	73,648.34	1.07 (1.06–1.09)	** < 0.001**
Age groups (years)				
12–29	6	20,884.64	1	
30–44	19	20,617.19	3.47 (1.38–8.71)	**0.008**
45–59	32	24,156.78	4.94 (2.06–11.82)	** < 0.001**
60–74	51	7989.73	22.65 (9.72–52.81)	** < 0.001**
Area deprivation index				
1–3 quintile (lowest deprivation)	50	39,147.68	1	
4 quintile	26	13,429.17	1.51 (0.94–2.43)	0.087
5 quintile (highest deprivation)	19	11,656.27	1.27 (0.75–2.16)	0.370
Missing	13	9415.22	1.07 (0.58–1.96)	0.839
Marital status				
Married	39	20,964.02	1	
Unmarried	28	32,159.99	0.45 (0.28–0.74)	**0.001**
Separated/divorced	17	9947.09	0.95 (0.54–1.68)	0.862
Widowed	12	1674.07	3.80 (1.99–7.27)	** < 0.001**
Missing	12	8903.17	0.70 (0.37–1.34)	0.286
Education				
University/High school	26	16,463.83	1	
Middle school	46	33,746.21	0.87 (0.53–1.40)	0.556
Elementary school	20	8696.26	1.40 (0.79–2.51)	0.255
None	4	5535.84	0.37 (0.13–1.07)	0.068
Missing	12	9206.2	0.77 (0.39–1.52)	0.444
Family composition				
Couples with children	26	29,885.42	1	
Couples without children	24	8945.8	3.25 (1.86–5.66)	** < 0.001**
Single parent	20	10,926.96	2.12 (1.19–3.80)	**0.011**
Single	24	13,055.24	2.25 (1.29–3.92)	**0.004**
Other^a^	2	1343.98	1.81 (0.43–7.63)	0.418
Missing	12	9490.94	1.47 (0.74–2.91)	0.272
Psychiatric disorders				
None/Other disorders^b^	36	36,602.45	1	
Schizophrenia	40	22,235.15	1.85 (1.18–2.90)	**0.007**
Mood disorders^c^	32	14,810.74	2.11 (1.31–3.39)	**0.002**
Malignant neoplasms				
No	76	69,209.33	1	
Yes	32	4439.01	6.60 (4.37–9.99)	** < 0.001**
Neurological disorders				
No	82	62,185.65	1	
Yes	26	11,462.69	1.79 (1.15–2.78)	**0.010**
Diabetes mellitus				
No	91	69,357.38	1	
Yes	17	4290.96	3.13 (1.86–5.25)	** < 0.001**
Cardiovascular diseases				
No	67	59,062.47	1	
Yes	41	14,585.87	2.53 (1.71–3.73)	** < 0.001**
Chronic obstructive pulmonary disease				
No	90	69,198.76	1	
Yes	18	4449.58	3.11 (1.87–5.15)	** < 0.001**
Dorsopathies				
No	83	55,851.4	1	
Yes	25	17,796.94	0.97 (0.62–1.52)	0.897
Intracranial injury/Skull fracture				
No	86	63,036.72	1	
Yes	22	10,611.62	1.57 (0.98–2.50)	0.061

Statistically significant results are marked in bold

*PY* person-years, *HR* hazard ratios, *CI* confidence interval, *P* *p*-value

^a^Other: families with two or more nucleus or other relatives

^b^Other disorders: personality disorder, drug and alcohol dependence, anxiety disorder, adjustment disorder, dementia and other mental disorders due to organic condition

^c^Mood disorders: bipolar disorder, depressive disorder

In the multiple Cox regression model, male suicide attempters had 86% higher risk of premature death than females (HR 1.86, 95%CI 1.24–2.78). The risk of death was associated with age, with 6% increased risk for each year of increase in age (HR 1.06, 95%CI 1.04–1.08). High deprivation index of the residence area (4th quintile) was associated with 71% increased risk of mortality compared to low deprivation (1st–3rd quintile) (*p* = 0.037). Single-parent family was a significant predictor of death as compared to family defined as couples with children, and the risk was doubled (HR 2.48, 95%CI 1.36–4.53). In sensitivity analysis including marital status in the model instead of family composition, widowed patients were associated with twofold higher risk of death (HR 2.44, 95%CI 1.22–4.90) (data not shown). Mood disorders predicted 72% increased risk of death as compared to no or other psychiatric diagnosis (*p* = 0.037). The diagnosis of intracranial injury or skull fracture and malignant neoplasms were associated with 1.67–2.71 times greater mortality (*p* = 0.050, *p* < 0.001, respectively), whilst the other diseases did not reach significance level (Table 3).

**Table 3:** Risk factors for all-cause, natural-cause and unnatural-cause death: multivariate Cox regression models

Characteristics	All-cause death (2010–2020) *N* = 1489			Natural-cause death (2010–2018) *N* = 1082			Unnatural-cause death (2010–2018) *N* = 1073	
	Adj HR (95% CI)	*P*	*n*/*N*	Adj HR (95% CI)	*P*	*n*/*N*	Adj HR (95% CI)	*P*
Gender (ref: female)			19/579			11/571		
Male	1.86 (1.24–2.78)	**0.003**	25/503	1.41 (0.74–2.70)	0.300	24/502	2.51 (1.21–5.24)	**0.014**
Age (years)	1.06 (1.04–1.08)	** < 0.001**	44/1082	1.08 (1.05–1.12)	** < 0.001**	35/1073	1.03 (1.00–1.06)	**0.051**
Area deprivation index (ref: 1–3 quintile)			18/583			18/583		
4 quintile	1.71 (1.03–2.83)	**0.037**	11/197	1.93 (0.79–4.68)	0.147	8/194	1.53 (0.64–3.65)	0.333
5 quintile (highest deprivation)	1.29 (0.74–2.25)	0.364	8/164	1.39 (0.55–3.55)	0.487	4/160	0.81 (0.27–2.45)	0.704
Missing	5.18 (0.80–33.39)	0.084	7/138	1.97 (0.02–210.91)	0.776	5/136	8.44 (1.07–66.88)	0.043
Family composition (ref: couples with children)			6/451			10/455		
Couples without children	1.50 (0.83–2.71)	0.178	15/131	3.05 (1.09–8.49)	**0.033**	4/120	1.01 (0.30–3.38)	0.990
Single parent	2.48 (1.36–4.53)	**0.003**	8/166	4.68 (1.49–14.71)	**0.008**	7/165	2.24 (0.84–5.98)	0.108
Single	1.03 (0.58–1.82)	0.924	8/177	1.18 (0.39–3.62)	0.768	9/178	1.32 (0.51–3.39)	0.564
Other^a^	2.79 (0.65–11.97)	0.166	0/18	–		1/19	3.08 (0.38–24.84)	0.290
Missing	0.36 (0.05–2.56)	0.309	7/139	3.10 (0.03–347.88)	0.638	4/136	0.17 (0.02–1.68)	0.128
Psychiatric disorders (ref: none/other disorders^b^)			14/535			13/534		
Schizophrenia	1.43 (0.90–2.30)	0.134	16/326	1.17 (0.53–2.58)	0.638	12/322	1.25 (0.55–2.83)*	0.600
Mood disorders^c^	1.72 (1.03–2.85)	**0.037**	14/221	2.15 (0.93–4.99)	0.074	10/217	1.74 (0.73–4.13)*	0.211
Malignant neoplasms (ref: no)			20/1010			32/1022		
Yes	2.71 (1.69–4.33)	** < 0.001**	24/72	5.40 (2.68–10.87)	** < 0.001**	3/51	1.14 (0.32–4.02)	0.836
Neurological disorders (ref: no)			32/918			27/913		
Yes	1.09 (0.67–1.77)	0.741	12/164	1.55 (0.74–3.27)	0.248	8/160	1.34 (0.56–3.17)	0.510
Diabetes Mellitus (ref: no)			36/1013			31/1008		
Yes	1.69 (0.95–2.99)	0.072	8/69	3.14 (1.31–7.54)	**0.011**	4/65	1.48 (0.47–4.63)	0.504
Cardiovascular diseases (ref: no)			26/876			26/876		
Yes	0.85 (0.54–1.32)	0.460	18/206	0.85 (0.43–1.66)	0.635	9/197	0.70 (0.29–1.71)	0.439
Chronic obstructive pulmonary disease (ref: no)			35/1013			31/1009		
Yes	1.54 (0.89–2.69)	0.124	9/69	1.27 (0.53–3.05)	0.586	4/64	1.55 (0.51–4.75)*	0.444
Intracranial injury/Skull fracture (ref: no)			38/932			28/922		
Yes	1.67 (1.00–2.79)	**0.050**	6/150	0.58 (0.21–1.60)	0.291	7/151	1.50 (0.62–3.59)	0.365

Statistically significant results are marked in bold

*Adj HR* adjusted hazard ratios, *CI* confidence interval, *P* *p*-value, *n/N* number of deaths/Number of patients in the group

*Statistical test showed violation of proportional hazard for these factors. These results should be taken with caution

^a^Other: families with two or more nucleus or other relatives

^b^Other disorders: personality disorder, drug and alcohol dependence, anxiety disorder, adjustment disorder, dementia and other mental disorders due to organic condition

^c^Mood disorders: bipolar disorder, depressive disorder

### Cox regression models for natural- and unnatural-cause mortality

Age was a significant predictor of natural-cause mortality, with 8% increased risk for each year of increase in age (HR 1.08, 95%CI 1.05–1.12). Living in couples without children and single-parent family was associated with threefold (HR 3.05, 95%CI 1.09–8.49) and fourfold (HR 4.68, 95%CI 1.49–14.71) increased risk of mortality compared to couples with children, respectively. As regards chronic diseases, malignant neoplasms (HR 5.40, 95%CI 2.68–10.87) and diabetes mellitus (HR 3.14, 95%CI 1.31–7.54) were significant predictors of natural death (Table 3).

When looking at unnatural-cause mortality, males had twofold higher risk of death compared to females (HR 2.51, 95%CI 1.21–5.24). Age was associated with 3% increased risk of death for each year of increase in age (HR 1.03, 95%CI 1.00–1.06). All other variables did not reach statistical significance. In sensitivity analysis including marital status instead of family composition in the model, widowed patients had fivefold greater risk of death (HR 5.00, 95%CI 1.24–20.11) (data not shown).

## Discussion

We conducted a cohort study by linking health and administrative data of the Piedmont Region following prospectively 1489 patients who were admitted to hospital or ED for suicide attempt between 2010 and 2020. To our knowledge, this is the first study to investigate risk factors for mortality in a cohort of hospitalized suicide attempters over 11-year span in Italy. Our study extends prior findings by adding new evidence from the South-European context. In this study, male gender, older age, high area deprivation index, single-parent family, comorbidity with mood disorders, malignant neoplasms and intracranial injury or skull fracture were predictors of all-cause death following the first hospital admission for attempted suicide. Older age, living in couples without children and single-parent family, being affected by malignant neoplasms and diabetes mellitus predicted natural-cause death, whereas gender and older age increased the risk of unnatural-cause death. The risk of death was highest during the first 12 months after suicide attempt, and remained elevated for many years afterwards.

The mean age at first episode of suicide attempt was in the range with previous studies [3, 17, 41, 42]. About one third of all suicide attempts occurred in adolescents and young adults, similarly to one fourth reported in a 9-year follow-up study in Belgium [43]. Consistently with prior studies, a greater proportion of younger females and older males attempted suicide [44, 45]. Our study also confirms earlier findings that psychiatric disorders and physical illnesses co-occur commonly in suicide attempters [3, 7, 21, 22, 24, 25, 28, 33, 35–37, 46–49].

During the observation period, 7.3% of subjects died, similar to previously observed 6.4% in Canadian and 8.7% in USA cohorts [9, 10], but lower than 12.1% reported in the Italian cohort by Pavarin and colleagues [16]. Although the risk for suicide and non-suicidal mortality persists for many years following the first episode, it is particularly pronounced shortly after the attempt episode [1–3, 6, 7, 10–13, 16, 17, 20, 21, 50]. In our study, 45% of deaths occurred in the first 12 months: this result points out that the period following the attempt episode is the most critical, and suggests that integrated and intense prevention and care interventions should be implemented in this period.

In the adjusted regression model, several characteristics emerged as significant predictors of mortality following suicide attempt. It is already well established that male gender is a significant predictor of mortality, particularly for suicide, among suicide attempters [1, 4–7, 9, 16, 18, 20, 32, 33]. Consistently, also in our study, the survival time was significantly lower for males than females [1, 2, 4, 5, 33, 50]. This may be attributable to a higher tendency of males to use violent methods and their higher intention to die, a relevant risk factors for suicide particularly within the first months after the attempt [3, 5–7, 13–15, 18, 31, 34, 37, 42, 51]. Moreover, males in our cohort had more alcohol and drug related problems than female suicide attempters, a behaviour that may contribute to violent causes of death [3, 4, 6, 7, 20, 42, 51]. This result is similar to that observed in the Finnish cohort by Suominen and colleagues [4]. Moreover, in our study, a greater proportion of males was diagnosed with intracranial injury or skull fracture, conditions that may act as a proxy of violent suicide methods and therefore be linked to a higher risk of death for unnatural causes.

In agreement with earlier studies, the risk of death increased with the increase of age at the time of suicide attempt [1, 5–7, 9, 16, 18, 20, 31–33, 49], possibly due to higher prevalence of health problems in older patients [31, 35]. Indeed, in our study, the prevalence of physical and mental illnesses increased proportionally with increased age (data not shown). Moreover, with advanced age the proportion of widowed patients increases, which may result in loneliness and living alone, recognized risk factors for death in general population and in suicide attempters [6, 11, 20]. Indeed, the association of widowed condition with the risk of all-cause and unnatural-cause deaths was also observed in our study (data not shown).

Suicide attempters living in highly deprived areas were at 71% greater risk of mortality as compared to those living in less deprived areas, congruently with what observed elsewhere using income and education level as indicators of socioeconomic status [14, 32]. Patients of high deprived areas may be at higher risk of psychiatric disorders or physical illnesses, have limited access to social and medical resources, poor social support, bad diet habits, substance use related problems and poor skills to cope with adverse life situations compared to those of more advantaged areas. These characteristics may therefore exacerbate the negative outcomes of a frail condition as suicide attempt. Particular attention should be paid to patients of low socioeconomic level after suicide attempt.

The importance and mechanisms of family composition that may impact the effect of attempted suicide on mortality have been understudied. In our study, living in single-parent family was associated with an increased risk of all-cause and natural-cause mortality compared to living in couples with children. This is expected since, single parents may experience stressful life events, economic disadvantage, and social and psychological pressure to a greater extent than two-parent families. However, in a previous Finnish study, an increased risk of natural- and violent-cause mortality was observed among single-parent families of general population, but not among suicide attempters [11].

Accounting for all other variables included in the model, being affected by mood disorders was an independent risk factor for all-cause mortality, similarly to what observed in other studies [3, 7, 9, 13, 21, 33, 37]. However, although the direction of effect was consistent, the statistical significance was not reached when the impact on natural and unnatural deaths was tested separately, probably due to limited statistical power of the restricted sample. Moreover, due to violation of proportional hazard assumption, the results of the model assessing unnatural causes of death should be taken with caution. Nevertheless, these findings point out the need to apply particular attention in diagnosis of concurrent psychiatric disorders, and in prescribing effective treatment in order to prevent mortality risk.

As regards physical comorbidities, an increased risk of all-cause and natural-cause deaths was observed for patients suffering from malignant neoplasms and diabetes mellitus. Physical illnesses are significant predictors of death among suicide attempters [2, 5], especially in case of chronic and severe conditions. Moreover, it is recognised that suicidal patients with chronic conditions are more depressed [35], which may be associated with severe health outcomes. Finally, intracranial injury or skull fracture was also a significant predictor of all-cause mortality, a diagnosis that may be related to suicide attempt method itself, e.g. violent attempts could lead to serious traumatic injuries, and in these cases the risk of death can be higher.

This study has a number of strengths. The sample included all episodes of suicide attempts referred from hospitals and ED in the region, irrespective of the method used. Several information on the study subjects were collected from administrative and health registers ensuring richness of the data and investigation of both sociodemographic factors and comorbidities. Completeness of the mortality data was high. However, the study results should be considered also in a light of some limitations. The suicide attempts are potentially underestimated due to misclassified and underreported cases, i.e. those not accessing ED or hospital, and those registered with other diagnoses, e.g. accidents. So, the sample may not be fully representative of all suicide attempts in the regional catchment area but biased toward the most seriously affected cases. We did not have the information on cause-specific mortality for the whole timeline of the cohort, but only until 2018. Therefore, Cox regression models assessing natural and unnatural causes of death were run on restricted samples, possibly resulting in a loss of statistical power for some factors. Moreover, the comparison of results across studies may be challenging due to the ambiguous and inconsistent definition of suicide attempt sometimes including self-injurious acts with and without suicidal intent. Finally, the study would deserve a longer follow-up.

In conclusion, our results showed a risk of mortality particularly high within the first year after admission to hospital or ED for attempted suicide. Male gender, increased age, high area deprivation index, single-parent family, mood disorders, malignant neoplasm, diabetes mellitus and intracranial injury or skull fracture were important risk factors for mortality after suicide attempt. Focusing on these predictors can help to identify high-risk groups, inform clinicians on medical and psychiatric needs of suicidal patients, and plan outpatient care following hospital discharge. Our findings urge the need to design strategies for the assistance and care of these patients at long term in order to reduce the unfavourable outcomes.

## Data Availability

Not applicable.

## References

[1] Ostamo A, Lönnqvist J (2001). Excess mortality of suicide attempters. Soc Psychiatry Psychiatr Epidemiol.

[2] Suokas J, Suominen K, Isometsä E, Ostamo A, Lönnqvist J (2001). Long-term risk factors for suicide mortality after attempted suicide–findings of a 14-year follow-up study. Acta Psychiatr Scand.

[3] Skogman K, Alsén M, Ojehagen A (2004). Sex differences in risk factors for suicide after attempted suicide-a follow-up study of 1052 suicide attempters. Soc Psychiatry Psychiatr Epidemiol.

[4] Suominen K, Isometsä E, Haukka J, Lönnqvist J (2004). Substance use and male gender as risk factors for deaths and suicide–a 5-year follow-up study after deliberate self-harm. Soc Psychiatry Psychiatr Epidemiol.

[5] Suominen K, Isometsä E, Ostamo A, Lönnqvist J (2004). Level of suicidal intent predicts overall mortality and suicide after attempted suicide: a 12-year follow-up study. BMC Psychiatry.

[6] Christiansen E, Jensen BF (2007). Risk of repetition of suicide attempt, suicide or all deaths after an episode of attempted suicide: a register-based survival analysis. Aust N Z J Psychiatry.

[7] Haukka J, Suominen K, Partonen T, Lönnqvist J (2008). Determinants and outcomes of serious attempted suicide: a nationwide study in Finland, 1996–2003. Am J Epidemiol.

[8] World Health Organization (2014). Preventing suicide: a global imperative.

[9] Finkelstein Y, Macdonald EM, Hollands S, Sivilotti ML, Hutson JR, Mamdani MM, Koren G, Juurlink DN, Canadian Drug Safety and Effectiveness Research Network (CDSERN) (2015). Risk of suicide following deliberate self-poisoning. JAMA Psychiat.

[10] Bostwick JM, Pabbati C, Geske JR, McKean AJ (2016). Suicide attempt as a risk factor for completed suicide: even more lethal than we knew. Am J Psychiatry.

[11] Mäki NE, Martikainen PT (2017). Premature mortality after suicide attempt in relation to living arrangements. A register-based study in Finland in 1988–2007. Eur J Public Health.

[12] Jokinen J, Talbäck M, Feychting M, Ahlbom A, Ljung R (2018). Life expectancy after the first suicide attempt. Acta Psychiatr Scand.

[13] Probert-Lindström S, Berge J, Westrin Å, Öjehagen A, Skogman Pavulans K (2020). Long-term risk factors for suicide in suicide attempters examined at a medical emergency in patient unit: results from a 32-year follow-up study. BMJ Open.

[14] Holley HL, Fick G, Love EJ (1998). Suicide following an inpatient hospitalization for a suicide attempt: a Canadian follow-up study. Soc Psychiatry Psychiatr Epidemiol.

[15] Gibb SJ, Beautrais AL, Fergusson DM (2005). Mortality and further suicidal behaviour after an index suicide attempt: a 10-year study. Aust N Z J Psychiatry.

[16] Pavarin RM, Fioritti A, Fontana F, Marani S, Paparelli A, Boncompagni G (2014). Emergency department admission and mortality rate for suicidal behaviour. A follow-up study on attempted suicides referred to the ED between January 2004 and December 2010. Crisis.

[17] Parra-Uribe I, Blasco-Fontecilla H, Garcia-Parés G, Martínez-Naval L, Valero-Coppin O, Cebrià-Meca A, Oquendo MA, Palao-Vidal D (2017). Risk of re-attempts and suicide death after a suicide attempt: a survival analysis. BMC Psychiatry.

[18] Vuagnat A, Jollant F, Abbar M, Hawton K, Quantin C (2020). Recurrence and mortality 1 year after hospital admission for non-fatal self-harm: a nationwide population-based study. Epidemiol Psychiatr Sci.

[19] Demesmaeker A, Chazard E, Hoang A, Vaiva G, Amad A (2022). Suicide mortality after a nonfatal suicide attempt: a systematic review and meta-analysis. Aust N Z J Psychiatry.

[20] Nordentoft M, Breum L, Munck LK, Nordestgaard AG, Hunding A, Laursen Bjaeldager PA (1993). High mortality by natural and unnatural causes: a 10 year follow up study of patients admitted to a poisoning treatment centre after suicide attempts. BMJ.

[21] Tidemalm D, Långström N, Lichtenstein P, Runeson B (2008). Risk of suicide after suicide attempt according to coexisting psychiatric disorder: Swedish cohort study with long term follow-up. BMJ.

[22] Bernal M, Haro JM, Bernert S, Brugha T, de Graaf R, Bruffaerts R, Lépine JP, de Girolamo G, Vilagut G, Gasquet I, Torres JV, Kovess V, Heider D, Neeleman J, Kessler R, Alonso J, ESEMED/MHEDEA Investigators (2007). Risk factors for suicidality in Europe: results from the ESEMED study. J Affect Disord.

[23] Bertolote JM, Fleischmann A, De Leo D, Bolhari J, Botega N, De Silva D, Tran Thi Thanh H, Phillips M, Schlebusch L, Värnik A, Vijayakumar L, Wasserman D (2005). Suicide attempts, plans, and ideation in culturally diverse sites: the WHO SUPRE-MISS community survey. Psychol Med.

[24] Nock MK, Borges G, Bromet EJ, Alonso J, Angermeyer M, Beautrais A, Bruffaerts R, Chiu WT, de Girolamo G, Gluzman S, de Graaf R, Gureje O, Haro JM, Huang Y, Karam E, Kessler RC, Lepine JP, Levinson D, Medina-Mora ME, Ono Y, Posada-Villa J, Williams D (2008). Cross-national prevalence and risk factors for suicidal ideation, plans and attempts. Br J Psychiatry.

[25] Borges G, Nock MK, Haro Abad JM, Hwang I, Sampson NA, Alonso J, Andrade LH, Angermeyer MC, Beautrais A, Bromet E, Bruffaerts R, de Girolamo G, Florescu S, Gureje O, Hu C, Karam EG, Kovess-Masfety V, Lee S, Levinson D, Medina-Mora ME, Ormel J, Posada-Villa J, Sagar R, Tomov T, Uda H, Williams DR, Kessler RC (2010). Twelve-month prevalence of and risk factors for suicide attempts in the World Health Organization World Mental Health Surveys. J Clin Psychiatry.

[26] Kõlves K, Vecchiato T, Pivetti M, Barbero G, Cimitan A, Tosato F, De Leo D (2011). Non-fatal suicidal behaviour in Padua, Italy, in two different periods: 1992–1996 and 2002–2006. Soc Psychiatry Psychiatr Epidemiol.

[27] Preti A (2012). Trends in suicide case fatality in Italy, 1983–2007. Psychiatry Res.

[28] Olfson M, Blanco C, Wall M, Liu SM, Saha TD, Pickering RP, Grant BF (2017). National trends in suicide attempts among adults in the United States. JAMA Psychiat.

[29] Twenge JM, Cooper AB, Joiner TE, Duffy ME, Binau SG (2019). Age, period, and cohort trends in mood disorder indicators and suicide-related outcomes in a nationally representative dataset, 2005–2017. J Abnorm Psychol.

[30] Bornheimer LA, Wang K, Zhang A, Li J, Trim EE, Ilgen M, King CA (2022). National trends in non-fatal suicidal behaviors among adults in the USA from 2009 to 2017. Psychol Med.

[31] Tidemalm D, Beckman K, Dahlin M, Vaez M, Lichtenstein P, Långström N, Runeson B (2015). Age-specific suicide mortality following non-fatal self-harm: national cohort study in Sweden. Psychol Med.

[32] Han B, Kott PS, Hughes A, McKeon R, Blanco C, Compton WM (2016). Estimating the rates of deaths by suicide among adults who attempt suicide in the United States. J Psychiatr Res.

[33] Chen HM, Hung TH, Chou SY, Tsai CS, Su JA (2016). Three-year mortality rate of suicide attempters in consultation-liaison service. Int J Psychiatry Clin Pract.

[34] Olfson M, Wall M, Wang S, Crystal S, Gerhard T, Blanco C (2017). Suicide following deliberate self-harm. Am J Psychiatry.

[35] De Leo D, Scocco P, Marietta P, Schmidtke A, Bille-Brahe U, Kerkhof AJ, Lonnqvist J, Crepet P, Salander-Renberg E, Wasserman D, Michel K, Bjerke T (1999). Physical illness and parasuicide: evidence from the European Parasuicide Study Interview Schedule (EPSIS/WHO-EURO). Int J Psychiatry Med.

[36] Scocco P, de Girolamo G, Vilagut G, Alonso J (2008). Prevalence of suicide ideation, plans, and attempts and related risk factors in Italy: results from the European Study on the Epidemiology of Mental Disorders-World Mental Health study. Compr Psychiatry.

[37] Runeson B, Haglund A, Lichtenstein P, Tidemalm D (2016). Suicide risk after nonfatal self-harm: a national cohort study, 2000–2008. J Clin Psychiatry.

[38] Istituto Nazionale di Statistica (ISTAT) (2019) Annuario Statistico Italiano 2019. Roma

[39] Rosano A, Pacelli B, Zengarini N, Costa G, Cislaghi C, Caranci N (2020). Aggiornamento e revisione dell’indice di deprivazione italiano 2011 a livello di sezione di censimento. Epidemiol Prev.

[40] Corporation S (2019). Stata Statistical Software: Release 16.

[41] Slama F, Courtet P, Golmard JL, Mathieu F, Guillaume S, Yon L, Jollant F, Mission H, Jaussent I, Leboyer M, Bellivier F (2009). Admixture analysis of age at first suicide attempt. J Psychiatr Res.

[42] Monnin J, Thiemard E, Vandel P, Nicolier M, Tio G, Courtet P, Bellivier F, Sechter D, Haffen E (2012). Sociodemographic and psychopathological risk factors in repeated suicide attempts: gender differences in a prospective study. J Affect Disord.

[43] De Munck S, Portzky G, Van Heeringen K (2009). Epidemiological trends in attempted suicide in adolescents and young adults between 1996 and 2004. Crisis.

[44] Iribarren C, Sidney S, Jacobs DR, Weisner C (2000). Hospitalization for suicide attempt and completed suicide: epidemiological features in a managed care population. Soc Psychiatry Psychiatr Epidemiol.

[45] Blasco-Fontecilla H, Alegria AA, Delgado-Gomez D, Legido-Gil T, Saiz-Ruiz J, Oquendo MA, Baca-Garcia E (2012). Age of first suicide attempt in men and women: an admixture analysis. ScientificWorldJournal.

[46] Goodwin RD, Marusic A, Hoven CW (2003). Suicide attempts in the United States: the role of physical illness. Soc Sci Med.

[47] Irigoyen M, Porras-Segovia A, Galván L, Puigdevall M, Giner L, De Leon S, Baca-García E (2019). Predictors of re-attempt in a cohort of suicide attempters: a survival analysis. J Affect Disord.

[48] Stickley A, Koyanagi A, Ueda M, Inoue Y, Waldman K, Oh H (2020). Physical multimorbidity and suicidal behavior in the general population in the United States. J Affect Disord.

[49] Hirot F, Ali A, Azouvi P, Naddaf A, Huas C, Guillaume S, Godart N (2022). Five-year mortality after hospitalisation for suicide attempt with a violent method. J Psychosom Res.

[50] Kuo CJ, Gunnell D, Chen CC, Yip PS, Chen YY (2012). Suicide and non-suicide mortality after self-harm in Taipei City, Taiwan. Br J Psychiatry.

[51] Zanone Poma S, Toniolo E, Grossi A, Pizzo R, Cocchio S, Baldo V (2013). Epidemiology of suicide attempts in psychiatric setting in Northern Italy. J Psychopathol.

